# A pilot study on measured insulin sensitivity and estimated glucose disposal rates in adults with type 1 diabetes and diabetic kidney disease

**DOI:** 10.1007/s12020-025-04503-5

**Published:** 2026-02-03

**Authors:** Johan R Simonsen, Daniel Gordin, Andrzej S Januszewski, Alicia J Jenkins, Daniël H van Raalte, Michael JB van Baar, Petter Bjornstad, Lena M Thorn, Per-Henrik Groop

**Affiliations:** 1 Folkhälsan Research Center, Biomedicum Helsinki, Helsinki, Finland; 2 Department of Nephrology, University of Helsinki and Helsinki University Hospital, Helsinki, Finland; 3 Research Program for Clinical and Molecular Metabolism, Faculty of Medicine, University of Helsinki, Helsinki, Finland; 4 Minerva Foundation Institute for Medical Research, Helsinki, Finland; 5 Helsinki Hypertension Centre of Excellence, University of Helsinki, Helsinki University Hospital, Helsinki, Finland; 6 Joslin Diabetes Center, Harvard Medical School, Boston, MA USA; 7 Sydney Pharmacy School, University of Sydney, Sydney, Australia; 8 NHMRC Clinical Trials Centre, University of Sydney, Sydney, Australia; 9 Baker Heart and Diabetes Institute, Melbourne, Australia; 10 Diabetes Center, Department of Internal Medicine, Amsterdam University Medical Centers, location VU University Medical Center, Amsterdam, The Netherlands; 11 Department of Medicine, Division of Metabolism, Endocrinology, and Nutrition, Seattle Children’s Research Institute, University of Washington Medicine Diabetes Institute (UWMDI), University of Washington School of Medicine, Washington, United States of America; 12 Department of General Practice and Primary Health Care, University of Helsinki, Helsinki University Hospital, Helsinki, Finland; 13 Department of Diabetes, Central Clinical School, Monash University, Melbourne, Australia

**Keywords:** Type 1 diabetes, Diabetic kidney disease, Insulin sensitivity, Diabetic complications.

## Abstract

**Purpose:**

Diabetic kidney disease (DKD) in type 1 diabetes has been shown to be strongly associated with insulin resistance, but this has not been previously explored using the euglycemic-hyperinsulinemic clamp. Therefore, we investigated insulin sensitivity in people with type 1 diabetes with and without DKD using M/I-values (mean glucose disposal rates [GDR]/mean plasma insulin) and compared GDRs to estimated GDR (eGDR)-formulae.

**Methods:**

In this pilot study, we studied 17 adult individuals with type 1 diabetes (ten with and seven without DKD) using euglycemic-hyperinsulinemic clamps and assessed correlations between GDR and eGDR values.

**Results:**

M/I-values were 62.5% lower in individuals with type 1 diabetes and DKD compared to those without DKD, albeit not statistically significant (0.16 ± 0.08 vs. 0.10 ± 0.08 mg/kg/min per mIU/L, *P* = 0.154). In the whole group (*n* = 17) eGDR by Williams et al. demonstrated the highest correlation with GDR (*r* = 0.35, *P* = 0.167), while eGDR by Januszewski et al. had the highest correlation in the DKD group (*n* = 10, *r* = 0.46, *P* = 0.177).

**Conclusion:**

Our pilot study suggests the possibility of increased insulin resistance in people with type 1 diabetes and DKD.

## Introduction

Insulin resistance is a key feature of type 2 diabetes, and an increasingly recognized feature of type 1 diabetes as well [1]. The gold standard of assessing insulin sensitivity is measuring the glucose disposal rate (GDR) in the euglycemic-hyperinsulinemic clamp. However, due to the required expertise, intensive nature and high costs of such studies this method is not feasible for large studies and mathematical formulae to estimate glucose disposal rates (eGDR) have been devised [2–4]. Diabetic kidney disease (DKD) has been shown to be strongly associated with insulin resistance [5–8], but to our knowledge no clamp studies have been undertaken to directly assess insulin sensitivity in individuals with type 1 diabetes and DKD. Furthermore, previously developed eGDR formulae in type 1 diabetes are based on individuals with mostly preserved kidney function. Therefore, in this pilot study, we investigated insulin resistance in individuals with type 1 diabetes with and without DKD and evaluated the correlations of three published eGDR formulas with measured GDR (mGDR).

## Materials and methods

The study is part of the Finnish Diabetic Nephropathy (FinnDiane) Study, a prospective, observational study comprising adult participants with type 1 diabetes (age at onset < 40 years and permanent insulin treatment started < 1 year after diagnosis) attending clinical study visits with 5-year intervals. We recruited FinnDiane participants, who at a study visit in the previous year met the following inclusion criteria: not having DKD (estimated glomerular filtration rate [eGFR] 90–125 mL/min per 1.73 m^2^ and urinary albumin excretion rate [AER] < 30 mg/24 h) or having DKD (diagnosis based on eGFR 30–89 mL/min per 1.73 m^2^ and AER ≥ 30 mg/24 h). eGFR was calculated using the Chronic Kidney Disease Epidemiology Collaboration (CKD-EPI 2021) formula [9]. The study was approved by the local ethics committee and conducted in accordance with the revised Declaration of Helsinki. All participants gave written informed consent.

Participants attended two clinical visits 2–4 weeks apart. At the first visit anthropometrics and blood pressure were measured, and blood samples collected. Body composition was measured by DXA (Lunar version 16; GE Healthcare, Wauwatosa, WI). The participant’s insulin regimen and blood glucose (BG) monitoring method, diet, and medications were assessed.

During the second visit an euglycemic-hyperinsulinemic clamp was performed. Participants abstained from alcohol, rigorous exercise (24 h), coffee (12 h) and collected 24 h urine samples prior to the visit. Clamps started at 7 am after an overnight fast. The prandial insulin dose was withheld. Intravenous cannulas were placed in both upper limbs for the infusions of 20% glucose and insulin, and blood sampling, respectively. Heating pads were applied for arterialization of venous blood. BG was measured every 5 min with a Biosen C-Line - Clinic/GP+-platform (EKF, Cardiff, United Kingdom). The insulin infusion (Insulin Aspart, NovoRapid^®^) was started at 40 mIU/(min⋅m^2^) with the goal to maintain a plasma glucose level of 5.0–5.5 mmol/l by adjusting the 20% glucose infusion rate. Clamps lasted for 240 min. During a 30 min steady-state phase at the end of the clamp, glucose infusion rates were measured for the GDR and calculation of M/I (mean GDR/mean plasma insulin). Insulin levels were measured using the Mercodia (Uppsala, Sweden) Iso-Insulin ELISA kit as per manufacturer’s instructions. The mGDR was compared with eGDR based on three published eGDR formulae: The Pittsburgh Epidemiology of Diabetes Complications (EDC) formula by Williams et al. (factors included: waist-hip ratio, presence of hypertension, HbA_1c_), the Coronary Artery Calcification in Type 1 Diabetes (CACTI) formula by Duca et al. (waist, total daily insulin dose per weight, triglycerides and diastolic blood pressure), and finally the eGDR-formula by Januszewski et al. (sex, age, high-density lipoprotein cholesterol [HDL-C], systolic and diastolic blood pressure, height, weight, waist and serum creatinine)[2–4].

Clinical characteristics are presented as mean ± SD, median (IQR), or frequency percentage. Differences between groups were tested using the Student’s t-test, Wilcoxon signed-rank test or Fisher’s exact test. Correlations between mGDR and eGDR were estimated using Spearman ρ correlation coefficients. All analyses were performed in R (R Core Team version 4.4.2, Vienna, Austria). Statistical significance was taken at *P* < 0.05.

## Results

We studied 17 Caucasian adults with type 1 diabetes (10 with DKD and seven with normal kidney function [Table 1]). Nine of the 10 participants with DKD had severe albuminuria (AER > 300 mg/24 h). No significant differences in age, diabetes duration, sex, baseline HbA_1c_, lipids were observed between the groups, but individuals with DKD had more central obesity compared to the group with retained kidney function (median waist to height ratio [IQR]: 0.57 vs. 0.51 (0.49–0.52), *P* = 0.043). All participants in the DKD group were on blockers of the renin-angiotensin-aldosterone system, while none of the participants in the other groups were treated with these or other hypertensive medications.

**Table 1:** Clinical characteristics of individuals with type 1 diabetes without or with diabetic kidney disease (DKD)

Parameter	All individuals with type 1 diabetes (*n* = 17)	Preserved kidney function (*n* = 7)	DKD (*n* = 10)	*P* (for difference between DKD and preserved kidney function)
Age, years	55.0 (44.9–57.0)	49.1 (43.6–52.5)	56.1 (55.2–60.7)	0.070
Sex (Female), n (%)	8 (47)	4 (57)	4 (40)	0.486
Diabetes duration, years	37.5 (28.6–47.2)	29.3 (22.8–40.9)	45.9 (30.0–50.0)	0.133
Usage of CSII, n (%)	7 (41)	4 (57)	3 (30)	0.298
Usage of MDI, n (%)	10 (59)	3 (43)	7 (70)	0.298
HbA_1c_, mmol/mol	53.2 ± 9.7	57.0 ± 3.4	50.5 ± 11.9	0.081
Creatinine, µmol/l	94 ± 29	113 ± 22	67 ± 5	< 0.001
eGFR, ml/min/1.73 m^2^	78.7 ± 23.1	102.9 ± 9.3	61.7 ± 10.5	< 0.001
Urinary AER, mg/24 h	303 (8–407)	0 (0–9)	401 (340–633)	< 0.001
Total cholesterol, mmol/l	3.4 (2.9–4.5)	4.2 (3.4–4.8)	3.1 (2.8–4.4)	0.282
HDL-cholesterol, mmol/l	1.3 (1.0–1.9)	1.7 (1.3–1.9)	1.2 (0.9–1.5)	0.133
LDL-cholesterol, mmol/l	2.0 (1.6–2.9)	2.2 (2.1–2.7)	1.6 (1.3–2.9)	0.171
Triglycerides, mmol/l	1.2 (0.8–1.5)	0.8 (0.7–1.1)	1.5 (1.1–1.8)	0.051
Systolic blood pressure, mmHg	143 ± 22	141 ± 23	144 ± 22	0.771
Diastolic blood pressure, mmHg	82 ± 12	84 ± 15	81 ± 10	0.591
Fat-percent	32.9 ± 0.1	31.0 ± 0.1	34.2 ± 0.1	0.400
Height (cm)	169 ± 13	171 ± 14	169 ± 14	0.770
Weight (kg)	77 ± 15	77 ± 18	77 ± 13	0.999
Waist (cm)	94 (83–103)	84 (80–93)	103 (87–104)	0.196
WHR	0.90 ± 0.11	0.85 ± 0.10	0.93 ± 0.11	0.131
BMI (kg/m^2^)	27.3 (25.6–28.0)	27.1 (24.9–27.7)	27.3 (25.8–27.9)	0.813
*Measured and estimated insulin sensitivity*				
GDR (mg/kg/min)	8.4 ± 2.4	8.8 ± 2.1	8.1 ± 2.6	0.516
GDR per lean mass (mg/kg/min)	12.5 ± 3.2	12.8 ± 2.8	12.2 ± 3.7	0.712
Insulin levels during steady state (µU/l)	67.9 (55.3–87.0)	68.3 (62.2-148.6)	56.4 (44.3–76.2)	0.172
Hypertension and/or use of antihypertensive medication	10 (59%)	0	10 (100%)	< 0.001
Total daily dose of insulin/bodyweight (IU/kg)	0.52 ± 0.17	0.44 ± 0.12	0.58 ± 0.18	0.090
M/I (mean GDR/mean plasma insulin, [mg/kg/min per mIU/L])	0.12 ± 0.07	0.16 ± 0.08	0.10 ± 0.08	0.154
eGDR by Williams formula, mg/kg/min	6.55 ± 2.51	8.93 ± 1.37	4.88 ± 1.57	< 0.001
eGDR by Duca formula, mg/kg/min	3.91 ± 1.65	4.87 ± 1.56	3.23 ± 1.41	0.046
eGDR by Januszewski formula, mg/kg/min	5.64 ± 4.64	9.96 ± 1.62	1.77 ± 1.88	< 0.001

CSII indicates continuous subcutaneous insulin infusion; MDI, multiple daily insulin injections; AER, albumin excretion rate; HbA1c, glycated hemoglobin; eGFR, estimated glomerular filtration rate; HDL, high-density lipoprotein cholesterol; LDL, low-density lipoprotein cholesterol; WHR, waist-hip ratio; BMI, body mass index; GDR, glucose disposal rate; eGDR, estimated glucose disposal rate. Data are presented as means ± standard deviation, medians (interquartile range), or percentages where appropriate

**Fig. 1:**
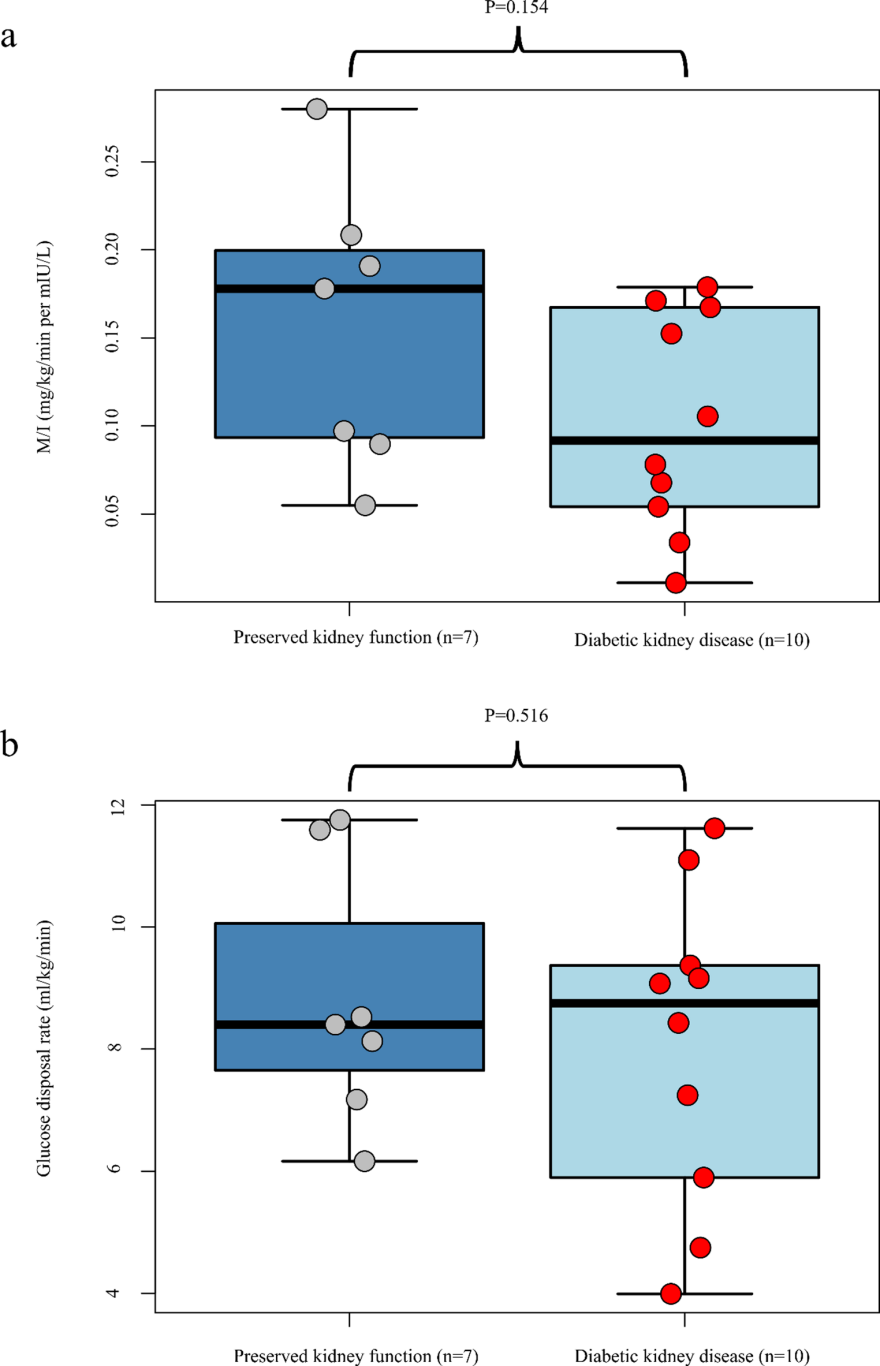
Insulin sensitivity in adults with type 1 diabetes with and without diabetic kidney disease (DKD). **a** Mean glucose disposal rate (GDR)/mean plasma insulin, mg/kg/min per µU/L insulin. **b** Measured GDR (mg/kg bodyweight/min). The box represents upper and lower quartile, the whiskers range, the line represents median with data points as dots

During clamp steady state, average BG was 5.4 mmol/l (SD 0.4), and the median insulin concentration was 66.6 µU/ml (IQR: 53.0-89.3). M/I-values were 62.5% lower in type 1 diabetes individuals with vs. without DKD, albeit not statistically significant (0.10 ± 0.08 vs. 0.16 ± 0.08 mg/kg/min per mIU/L, *P* = 0.154, Fig. 1a). Differences in mean [SD] GDR were smaller (8.8 [2.1] vs. 8.1 [2.6] mg/kg/min, *P* = 0.516). We also observed substantial variability in GDR in both groups, especially in the DKD group where GDR-values ranged from 4 to 12 mg/kg/min (Fig. 1b).

Comparing the previously published eGDR-formulae with our mGDR, the eGDR-formulae generally exhibited weak correlations: in all 17 participants the Spearman correlation coefficients between mGDR and eGDRs calculated were *r* = 0.35 (*P* = 0.167) for the Williams formula, *r* = 0.34 (*P* = 0.184) for the Duca formula, and *r* = 0.24 (*P* = 0.350) for the Januszewski formula. Similarly, in individuals without DKD, all formulae showed weak correlations: Williams, *r* = 0.30, *P* = 0.518; Duca, *r* = 0.05, *P* = 0.91; and Januszewski, *r*=-0.24, *P* = 0.613. In individuals with DKD all formulae showed modest correlations: Williams, *r* = 0.43, *P* = 0.222; Duca, *r* = 0.46, *P* = 0.184; and Januszewski, *r* = 0.46, *P* = 0.177. Interestingly, all three eGDR formulas predicted statistically significant differences in insulin sensitivity between the two groups, with lower sensitivity in those with vs. without DKD (Table 1).

## Discussion

Insulin resistance has been associated with chronic kidney disease [6,10]. To our knowledge it has never previously been investigated in people with type 1 diabetes and established DKD using the euglycemic-hyperinsulinemic clamp methodology. Herein we measured insulin sensitivity by this “gold-standard” technique and also assessed the performance of three eGDR formulae in 17 adults with type 1 diabetes with and without DKD. Individuals with DKD seemed more insulin resistant and had ≈ 63% lower insulin sensitivity (M/I) compared to those without DKD. mGDR was also lower in those with vs. without DKD, but again did not reach statistical significance in this pilot study. Interestingly, all three eGDR formulae predicted a significant difference in eGDR between the two groups. The Williams formula showed the best correlation in the whole group, while the Januszewski formula performed best in individuals with DKD.

Some limitations must be considered. Taking our small sample size into account, our pilot study likely lacked the statistical power to detect significant differences in insulin sensitivity between the groups. Furthermore, our sample set was quite heterogenous considering the large variability in GDR-values we observed across participants as well as the difference between the two groups in terms of chronic diabetes complications, which may further impede statistical power in our study. However, despite the lack of statistical significance we still believe the difference observed in insulin resistance between the two groups to be clinically relevant. The strengths of the study include carefully characterized participants and reliable GDR measurements. Notably, our participants with DKD also had quite advanced kidney disease as nine out of ten had severe macroalbuminuria and reduced eGFR. Therefore, the present study sheds some new light on insulin resistance in advanced diabetic kidney disease in type 1 diabetes, to our knowledge not previously explored. Based on the results of our pilot study we estimate that to have 90% power to show a statistically significant difference (two-sided *P* < 0.05) in clamp-measured insulin sensitivity between two groups of adults with type 1 diabetes with and without DKD at least 21 individuals per group would be required.

Regarding the difference in eGDRs and our mGDRs, it is of note that our participants differed from the other studies subjects, from which the three eGDR formulae were derived. Compared to the other studies (Williams, Duca, Januszewski), our participants were older and had a longer diabetes duration. The glycemic control in our participants, especially in the DKD group, was also fairly good and markedly better than in the Williams study. The varying correlations may also be explained by the fact that eGDR-values poorly correlate with both mGDRs as well as with each other [4]. As all participants were white it is unclear how the results apply to other ethnic populations.

To conclude, our study implies that there is a likely increased insulin resistance in adult individuals with type 1 diabetes and DKD relative to those without DKD. Euglycemic clamps in larger cohorts are needed to confirm this, and to elucidate which eGDR-formulas may best predict insulin sensitivity in these individuals.

## Data Availability

Individual-level data for the study participants are not publicly available because of the restrictions by the participant at the time of data collection.  Readers may propose collaboration to research the individual level data via correspondence with the lead investigator.

## Abbreviations

GDR: Glucose disposal rate (GDR)
eGDR: Estimate glucose disposal rates (eGDR)
DKD: Diabetic kidney disease (DKD)
mGDR: Measured GDR (mGDR)
FinnDiane: Finnish Diabetic Nephropathy Study (FinnDiane)
eGFR: Estimated glomerular filtration rate (eGFR)
AER: Albumin excretion rate (AER)
CKD-EPI 2021: Chronic Kidney Disease Epidemiology Collaboration 2021 formula (CKD-EPI 2021)
BG: Blood glucose (BG)
mean GDR/mean plasma insulin: M/I (mean GDR/mean plasma insulin)
HDL-C: High-density lipoprotein cholesterol (HDL-C)
LDL-C: Low-density lipoprotein cholesterol (LDL-C)
WHR: Waist-hip ratio (WHR)
BMI: Body mass index (BMI)
CSII: Continuous subcutaneous insulin infusion (CSII)
MDI: Multiple daily insulin injections (MDI)
